# Effects of Dietary Enrichment with Olive Cake on the Thyroid and Adrenocortical Responses in Growing Beef Calves

**DOI:** 10.3390/ani13132120

**Published:** 2023-06-26

**Authors:** Esterina Fazio, Arianna Bionda, Vincenzo Chiofalo, Deborah La Fauci, Cinzia Randazzo, Alessandra Pino, Paola Crepaldi, George Attard, Luigi Liotta, Vincenzo Lopreiato

**Affiliations:** 1 Dipartimento di Scienze Veterinarie, University of Messina, Viale Palatucci 13, 98168 Messina, Italy; esterina.fazio@unime.it (E.F.); vincenzo.chiofalo@corfilcarni.it (V.C.); deborah.lafauci@unime.it (D.L.F.); luigi.liotta@unime.it (L.L.); vincenzo.lopreiato@unime.it (V.L.); 2 Dipartimento di Scienze Agrarie e Ambientali—Produzione, Territorio, Agroenergia, University of Milano, Via Celoria 2, 20133 Milano, Italy; paola.crepaldi@unimi.it; 3 Consortium of Research for Meat Chain and Agrifood (CoRFilCarni), Viale Palatucci 13, 98168 Messina, Italy; 4 Department of Agriculture, Food and Environment, University of Catania, S. Sofia Street 100, 95123 Catania, Italy; cranda@unict.it (C.R.); alessandra.pino@unict.it (A.P.); 5 ProBioEtna SRL, Spin-Off of University of Catania, S. Sofia Street 100, 95123 Catania, Italy; 6 Department of Rural Sciences and Food Systems, University of Malta, 2080 Msida, Malta; george.attard@um.edu.mt

**Keywords:** beef cattle, thyroid hormones, cortisol, olive cake, by-products, circular economy, growth

## Abstract

**Simple Summary:**

The persistent demands for better understanding of the relationship between endocrine adaptation, BW, and productive performances from the perspective of both farmers and the market values of the beef carcass underline that further research on this main topic may be of great interest. In intensive beef production systems, the increase in the prices of feed ingredients has led to changes in the feed formulations, and agri-food wastes can be valorised by incorporating them in diet formulations. In this context, the use of olive cake, a by-product of the olive oil industry, in the formulation of animal feed has been linked with positive effects on the quality of final products, but no previous studies have been conducted to evaluate the thyroid and adrenocortical response to a diet supplemented with olive cake in young growing Limousine beef calves. The present study reveals that the inclusion of olive cake at levels of 10% or 15% in grower/finisher beef diet formulations does not have a detrimental effect on maintaining the physiological thyroid and adrenocortical hormones’ concentrations.

**Abstract:**

Agro-industrial by-products incorporated into livestock feed formulations can positively impact feed costs and promote a circular bio-economy. Italy produces significant amounts of olive cake (OC), a by-product of olive oil extraction, with the potential for incorporation into bovine diets. However, information on its effects on endocrine responses in growing beef calves is lacking. Forty-eight Limousines randomly allocated to dietary treatment (control or 10%-OC or 15%-OC inclusion) were segregated according to sex and body weight. Serum concentrations of TSH, thyroid hormones, and cortisol were measured on day 0, day 56, and at the end of the trial on day 147. Circulating TSH, total (T_3_, T_4_) and free (fT_3_, fT_4_) iodothyronines, and cortisol concentrations were all within the normal physiological ranges, with no significant effect imparted by diet. However, the diet × time interaction was significant for T_3_. The cortisol, T_3_, T_4_, and fT_4_ registered on day 147 were higher than those of day 56, and cortisol was higher in heifers than bulls. Final body weight was positively correlated with TSH and T_3_ and negatively with cortisol concentration. These findings suggest that the inclusion of OC at levels up to 15% in growing/finishing beef diets had no adverse effects on the calves’ thyroid and cortical status.

## 1. Introduction

In pursuit of a healthier lifestyle, consumers across Europe are changing lifestyle habits, including a tendency to choose healthier food options. Meat enriched with beneficial compounds, such as unsaturated fatty acids and bioactive molecules (e.g., anti-oxidants), falls within the category of healthier meats. These beneficial compounds are very often present in some of the by-products of industrial agro-industrial processes. Olive cake, a by-product of the olive oil pressing industry, has significant concentrations of these beneficial compounds. The use of olive cake in animal feed introduces substances with anti-oxidant and radical scavenging activities into the diets whilst increasing the concentrations of monounsaturated fatty acids within the animal products (meat and milk) intended for human consumption [1,2,3,4]. Italy has an estimated yearly production of 429,000 tons of olive oil [5]; hence, a significant amount of olive cake is readily available. The UE disciplinary “QS Sicilia” endorses the inclusion of agro-industrial by-products within a circular economy, and the inclusion of 10% or more olive cake in beef cattle feeds is permitted. In this context, the incorporation of agro-industrial by-products into livestock diet formulations represents a valid strategy for reducing the potential environmental impact from their disposal while at the same time improving the quality of the animal products [6,7]. Hence, the inclusion of olive cake in beef diets contributes to decreasing feed costs to the benefit of the farmer, an issue of particular relevance due to the current Ukraine war, which has resulted in huge increases in grain prices [8]; reducing environmental implications to the benefit of the general society; and producing healthier meat to satisfy consumer demands.

The complex intricacies involved in the interplay between the effects of dietary contents on the health, welfare, and production parameters of growing cattle have aroused great curiosity in the scientific community [9], so much so that the amount of research dedicated to the nutritional management of growing heifers has doubled over the past decade [10]. Olive cake has already been shown to have a high nutritional value, with significant amounts of fibre, non-starch polysaccharides, monounsaturated fatty acids, and bioactive molecules, such as phenolic compounds [11]. Recent studies indicate that these bioactive molecules are involved in several processes, including glucose and lipid metabolism and inflammation [12,13], while other studies on various species reported a stimulating effect from olive oil and leaf extracts on the concentration of thyroid hormones (THs) (as reviewed in [14]). These hormones are known to be involved in the growth of calves through the regulation of the basal, lipidic, and glycidic metabolism [15,16]. These THs are also known to participate in the central regulation of energy balance at the level of the hypothalamus [17]. Moreover, hypothalamic–pituitary–thyroid (HPT) axis activity is heavily responsive to nutritional inputs and dietary factors. In this context, the availability of nourishment, substrate diversity, and the diet’s caloric content and composition all have effects on the TH activity and on the peripheral metabolism [18,19].

The hypothalamic–pituitary–adrenal (HPA) axis and cortisol also have crucial roles to play in the regulation of energy balance, food intake, and body weight. Previous research has demonstrated the existence of positive correlations between blood levels of cortisol and environmental stressors, including individual temperament and dietary intake, frequency of dietary energy supplementation, and other nutrition factors [20,21,22]. For example, it has been reported that the amount of dietary protein, as well as dietary carbohydrate, can alter cortisol secretion and change the pattern of body fat accumulation [23]. However, the literature on the effects of single macronutrients on circulating cortisol concentrations in animals and humans is limited and contradictory [24,25,26,27,28].

To the best of our knowledge, no previous studies have been carried out to evaluate the impact of olive cake integration in calves’ diets on their thyroid and adrenocortical response. Thus, in view of the above, and given that, especially in the Mediterranean area, the incorporation of olive cake in beef diets is now being encouraged as a good strategy for sustainable animal husbandry and circular economy [2,29], this study aimed to investigate whether dietary enrichment with olive cake can induce an effect on the homeostasis status of thyroid and adrenocortical activities in growing beef animals. 

## 2. Materials and Methods

The experimental protocol was approved by the Ethical Committee of the Department of Veterinary Science, Messina University, Italy (code 041/2020). This research was in compliance with the guidelines set in the Italian and European regulations on animal welfare (Directive 2010/63/EU) and guidelines of good clinical practices [30].

### 2.1. Animal Management and Experimental Design

The experimental design of this study was previously described by Bionda et al. [29]. Briefly, a total of 48 young Limousine calves (24 non-castrated bulls and 24 heifers), aged 250 ± 20 days at the time of first sampling, were randomly assigned to one of three dietary treatment groups and segregated according to sex and body weight (BW). Each treatment group consisted of one pen with 8 bulls and another pen with 8 heifers for a total of two pens with 16 animals per treatment. The pens had straw deep-litter bedding and were large enough to allow for 4.5 m^2^/head. All animals were weighed at the commencement of the trial and again at the conclusion of the trial. Throughout the duration of this feeding trial, the caretaker and the supervising veterinarian reported that none of the animals showed any clinical signs of morbidity.

The three treatment concentrate diets were formulated as following: control group (CTR; 0% inclusion of olive cake), low-olive cake group (L-OC; 10% inclusion of olive cake), and high-olive cake group (H-OC; 15% inclusion of olive cake). All diets were formulated to have the same energy and protein content. The compositions and analytical analyses of all the treatment diets and of the OC used for the study are reported in Table 1. The approved UE disciplinary “QS Sicilia” recognizes that, as a strategy for the recovery of agro-industrial by-products, inclusion of olive cake in beef diets amounting to at least 10% of the formulation is permitted. The treatment diets were consumed twice daily (at 0800 and 1500 h) and all the animals had ad libitum access to straw and water.

### 2.2. Blood Samples and Analyses

The first blood sample was collected on day 0 of the trial (February, 250 ± 20 d of age), followed by a second collection on day 56 (April, 306 ± 20 d of age) and a final collection on day 147 (July, 397 ± 20 d of age). Blood was obtained from a venipuncture of the jugular vein and collected into 10 mL tubes containing clot activator and separating gel (Terumo Corporation, Tokyo, Japan). All blood collections were carried out at 0700 h from overnight-fasted animals so as to exclude any potential effect that the physiologic circadian rhythm may have had on the animals’ hormone concentrations. Once all blood samples were collected, animals were offered feed according to their assigned treatment group,

Blood samples were centrifuged for 10 min at 2000× *g* and the supernatant serum was collected and stored at −20 °C until further analysis. Serum thyroid-stimulating hormone (TSH) and free thyroxine (fT_4_) concentrations were assessed using a veterinary homologous, solid-phase, two-site chemiluminescent immunometric assay (Immulite^®^ 2000, Siemens Medical Solutions, Los Angeles, CA, USA). Serum total triiodothyronine (T_3_) and thyroxine (T_4_) and free triiodothyronine (fT_3_) concentrations were assessed using a human homologous, solid-phase, two-site chemiluminescent immunometric assay (Immulite 2000, Siemens Medical Solutions). All hormonal analyses were completed at the Veterinary Diagnostic Center BIOGENE (Catania, Italy). The relative assays were validated for linearity using cows’ serum prior to use according to the recommendations and procedures found in the manufacturer’s instructions. The intra- and interassay coefficients of variation (CVs) were the following: for TSH, 5.5% and 9.5% at TSH concentrations of 0.2 and 2.35 ng/mL; for T_3_, 12% and 5.5% at T_3_ concentrations of 73 ng/dL and 171 ng/dL; for fT_3_, 9.1% and 5.4% at fT_3_ concentrations of 3.2 pg/dL and 13 pg/dL; for T_4_, 11.1% and 5.6% at T_4_ concentrations of 1.8 μg/dL and 16 μg/dL; and for fT_4_, 3.0% and 10.2% at fT_4_ concentrations of 4.82 ng/dL and 0.51 ng/dL. The detection sensitivity of the assay was 0.01 ng/mL for TSH, 19 ng/dL for T_3_, 1.0 pg/mL for fT_3_, 0.3 μg/dL for T_4_, and 0.11 ng/dL for fT_4_ concentrations. Serum cortisol concentrations were measured using a homologous, solid-phase, two-site chemiluminescent immunometric assay (Immulite^®^ 2000, Siemens Medical Diagnostic Solutions, Los Angeles, CA, USA) according to the manufacturer’s instructions. Cortisol intra- and interassay CVs were 0.27% and 6.1, respectively. The sensitivity of the assay was 0.20 μg/dL.

The TH system and TH-mediated signalling are known to play pivotal roles in the control of substrate utilization and thus are involved in thermoregulatory processes to maintain body temperature [31]. However, the environmental temperature throughout the duration of this experiment varied on average between 17 and 21 °C, which was well within the thermoneutral comfort zone for the Limousine breed. Hence, the occurrence of the physiological processes that are usually triggered by environmental temperatures beyond either side of the animals’ thermoneutral zone was excluded in this study.

### 2.3. Statistical Analysis

Statistical analyses were performed using JMP version 16 (SAS Institute Inc., Cary, NC, USA). Appropriate descriptive variables were employed. The correlation between all the parameters was expressed using Pearson’s coefficient (r).

The blood parameters of interest (Y) were modelled as follows:

$$Y_{\mathrm{ijk}}=m+D+T+S+\mathrm{DT}+\mathrm{DS}+T_{0}+e$$

where m is the mean, D is the diet (CTR, L-OC, or H-OC), T is the time of sampling (day 56 or day 147 of the trial), S is the sex of the animal (male or female), DT is the interaction between the diet and the time of sampling, DS is the interaction between the diet and the sex, T_0_ is the covariate representing the parameter level at the beginning of the trial (before administering the supplemented concentrate), and e is a random residual. A logarithmic transformation was applied when necessary. When factors or interactions showed significance, the Tukey–Kramer post hoc test was used to identify the significantly different levels. Differences were considered to be statistically significant when *p* < 0.05.

## 3. Results

The serum concentrations of TSH, thyroid hormones, and cortisol for all the groups, as well as the results of the model, are presented in Table 2.

**Figure 1:**
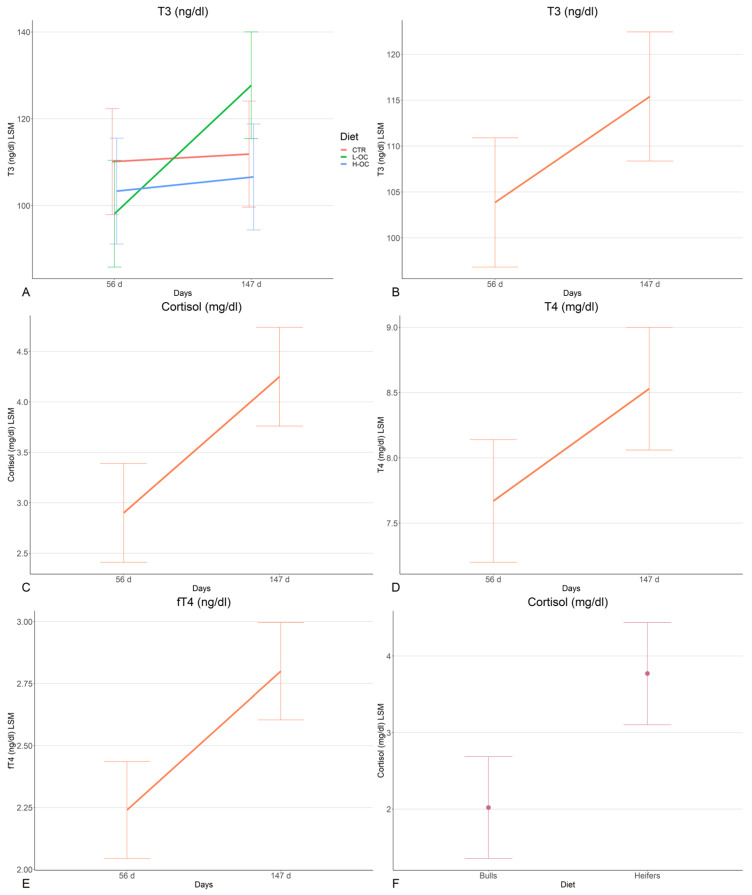
Representation of the significant model’s factors: the diet × time interaction for total triiodothyronine (T_3_, **A**); time for cortisol (**B**), T_3_ (**C**), total thyroxine (T_4_, **D**), and free thyroxine (fT_4_, **E**); and sex for cortisol (**F**).

No significant effects from the treatment diets on any of the measured hormones were observed (Table S1). However, the diet × time interaction did register as significant (*p* = 0.0470) for the T_3_ concentrations. The data indicate that, while there was only a minimal increase in mean T_3_ concentrations for the CTR and H-OC groups, there were higher levels in the L-OC group (from 98.13 ± 6.27 to 127.72 ± 6.27 mg/dL) (Figure 1A). The diet × sex effect registered a *p*-value close to the threshold of significance (*p* = 0.0566) for T_4_ concentration. Heifers and bulls had similar T_4_ concentrations in the CTR and L-OC groups, while T_4_ was registered as higher in females than males in the H-OC group. At the same time, the H-OC group showed the highest T_4_ level for heifers, while for the L-OC group, it was in bulls.

A significant effect for sampling time (day 56 vs. day 147) was observed, with the strongest being for cortisol (*p* = 0.0002), T_3_ (*p* = 0.0257), T_4_ (*p* = 0.0133), and fT_4_ (*p* = 0.0001) concentrations measured on day 147 when compared to day 56 (Figure 1B–E). Circulating cortisol and TH concentrations for the different sampling times, including day 0, are reported in Table 3.

The sex factor resulted in a significant effect on cortisol concentrations (*p* = 0.0003), with heifers (3.77 ± 0.34 mg/dL) registering higher values than bulls (2.02 ± 0.34 mg/dL) (Figure 1F).

Positive correlations were observed for T_3_ with T_4_ (r = 0.46; *p* < 0.0001), fT_4_ (r = 0.52; *p* < 0.0001), and fT_3_ (r = 0.38; *p* = 0.0002); T_4_ with fT_4_ (r = 0.63; *p* < 0.0001) and cortisol (r = 0.21; *p* = 0.0447); and cortisol with fT_4_ (r = 0.27; *p* = 0.0069).

The body weight at the end of the feeding trial was positively correlated with TSH (r = 0.3740; *p* = 0.0088) and T_3_ (r = 0.4590; *p* = 0.0010) but registered a negative correlation with the cortisol level (r = −0.5729; *p* ˂ 0.0001) that was measured on day 147. 

## 4. Discussion

The results obtained in the present study for serum TSH, total (T_3_, T_4_) and free (fT_3_, fT_4_) iodothyronines, and cortisol concentrations were comparable to those reported by different authors [32,33,34,35,36]. The most relevant outcomes of this study are the following:

(1) The absence of a significant effect from the dietary inclusion of OC on the concentrations of TSH, THs, and cortisol was noted;
(2) A significant effect for time was recorded with increasing concentrations of T_3_, T_4_, fT_4_, and cortisol, suggesting that demand from the animal on its energy reserves increases so as to meet the needs of increased metabolism due to growth;
(3) A sex-related difference was recorded for cortisol concentration but not for TSH or THs, with higher values in heifers than in bulls.

Previous studies have indicated that, in cattle, the concentrations of THs can be influenced through diet [37,38,39]: for example, the integration of tallow and cholesterol in the diet of young Holstein bull calves led to increased T_4_ and T_3_ concentrations and had the best growth response [40]. The present study found no significant effects from the OC-integrated diets on the TSH and TH levels. However, a significant effect for the diet × time interaction for T_3_ was observed, with a more pronounced increase in its concentrations from day 56 to day 147 in the L-OC group as opposed to the other two groups. These findings are in partial agreement with those obtained for buffaloes by Campanile et al. [19], where a high-energy diet resulted in an increase in T_3_ but not T_4_ or TSH when compared to a low-energy diet. The disagreement between the results of this study and those obtained by Campanile et al. [19] is probably due to the different species and experimental designs or the fact that all three diets administered in this study were formulated to be isoenergetic. The high- and low-energy diets used by Campanile et al. might have actually induced variations in the buffalo heifers’ metabolic homeostasis since research on cattle indicates that increased TH concentrations during daylight hours as opposed to night time can be attributed to higher feed intake during the daytime [41]. 

The contrasting results may suggest that other factors are at play, such as genetics and the growth rate, which could potentially account for the majority of the observed variation. The presence of positive correlations between concentrations of T_3_ and TSH and the body weight recorded at the conclusion of the trial (averaging 387 days of age), as well as the increases in T_3_, T_4_, and fT_4_ concentrations from day 56 to 147, corresponding to the finishing phase of body composition, offers supplementary support for the hypothesis that elevated growth rates in beef calves may exhibit a positive association with higher levels of circulating thyroid hormones [42]. These results substantiate the unique T_4_ and T_3_ effects on the metabolic adaptive responses to growth and energy balance in beef calves, similarly to what was recorded in growing foals [43] and other Brown calves [16]. The interaction between THs and growth is also corroborated by prior research indicating that growth hormones have a direct impact on the hypothalamic–hypophysis–thyroid axis in cattle, resulting in an increase in liver and kidney thyroxine-5’-monodeiodinase activity, as well as TH and TSH plasma concentrations [44]. 

Another interesting, although expected, finding was the strong correlation between T_3_:fT_3_ and T_4_:fT_4_. This was likely due to the homeostatic activity of the synthesis of both total and free iodothyronines as the animals approached the growth phase. The peripheral 5′-deiodinase activity is most likely subject to the same homeostatic control, as the expression of these enzymes changes during the lifetime of an individual in relation to the different needs of each organ and the needs due to ageing [45]. The positive correlation recorded between T_3_ and T_4_ agrees with observations suggesting that T_3_ should be considered the main biologically active thyroid hormone and that T_4_ needs be converted to T_3_ to trigger it into an active state [46]. Hence, the temporal variations in metabolic homeostasis were characterized by these positive and significant correlations consistently with previous reported results for growing foals, pregnant goats, and dairy cows, which indicated that changes in circulating total iodothyronines often follow fluctuations in the free form [43,47,48,49].

In this study, the sex of the animals registered as not significant for TSH and TH concentrations for heifers and bulls. This was in contrast to the study by Kahl and Bitman [42] that found gradual increases (38% and 96%) in T_4_ and T_3_ concentrations in both sexes during the period of 6 to 22 weeks of age, with higher values recorded in males than females. However, Kahl and Bitman’s model only accounted for a small portion of the variation in TH concentrations, which may have been influenced by other factors, such as genetics, physiological diurnal variation, ambient temperature, and seasonal photoperiod [50,51]. 

The influence of diet on cortisol secretion is well-documented [20,21,22,52]. Studies on sheep have found that the impact of glucocorticoids on feed intake and body weight depends on the hypothalamus’s set points, which are, in turn, affected by seasonal changes [53,54,55]. Glucocorticoids do not only affect body weight by modifying an animal’s feed intake but also alter the utilization of substrates, regardless of their impact on the animal’s energy balance. In fact, cortisol and/or corticosterone are considered to have lipolytic properties, and they mobilize fuel sources during times of stress [56,57]. Recent findings suggest that the extent of cortisol response may determine the metabolic consequences of stress. For instance, research on sheep has demonstrated that ewes with a high cortisol response tend to have higher adipose tissue accretion when exposed to a high-energy diet compared to those with a low cortisol response [58]. In ruminants, feed intake is controlled by a multifaceted mechanism involving the central nervous system, neurochemical and hormonal mechanisms, and the physical responses of the gastrointestinal tract [59,60]. However, in the case of beef cattle, the responsiveness of the HPA axis to stress does not seem to be a significant contributor to variations in feed efficiency in growing heifers [61]. In fact, beef heifers with divergent residual feed intake showed no significant differences in their physiological stress response to an exogenous bovine corticotropin-releasing hormone challenge [61]. The current study found no significant effect from diet on the cortisol changes since all treatment groups showed similar concentrations. It is possible to speculate that neither the frequency of dietary energy supplementation nor the level of inclusion of OC cause HPA axis hyperactivity since cortisol concentrations remained within the normal physiological ranges for this species throughout the trial. 

Although an increase in cortisol concentration was observed during the finishing phase, by the end of the trial period, the concentration was found to be negatively correlated with the final measured BW. This observation was in accordance with the findings of another experimental growth trial [62] where calves with higher cortisol levels showed reduced soft tissue and skeletal growth between the ages of 3 and 6 months. With regard to sex, the data obtained in this study indicate higher cortisol concentration in females, showing consistency with published data on Angus cattle [63,64,65]. Moreover, in another study on Bahman calves, sexual dimorphism was observed in an acute-phase response [64,66].

The interplay between thyroid hormones and cortisol in energy metabolism suggests that the thyroid and adrenocortical responses in finishing beef cattle serve as crucial endocrine signals for the initiation of energy utilization and the regulation of energy balance. Notably, TSH and T_3_ exert significant anabolic effects, as evidenced by their positive correlation with BW. On the other hand, the catabolic effect of cortisol was reaffirmed through its negative correlation with BW. However, the simultaneous effects of these hormones were balanced within their physiological ranges, irrespective of dietary supplementation with OC, as indicated by the positive correlations between BW, TSH, and T_3_ and the negative correlation between BW and cortisol.

## 5. Conclusions

The present study revealed that the inclusion of olive cake into grower/finisher beef diet formulations at levels of 10% and 15% did not have a detrimental effect on maintaining the physiological thyroid and adrenocortical hormones’ concentrations. Further studies on the relationship between comprehensive olive cake percentage responses and various traits, such as growth performance and feed intake, are warranted before recommending its use as a dietary supplement.

Moreover, the data obtained in this study show that each growing stage requires different endocrine strategies that may or may not have carryover effects on the animal performances. The persistent demands for knowledge about the relationship between endocrine adaptation, BW, and productive performances from the perspective of both farmers and the market value of the beef carcass underline that further research on this topic may be of great interest. The assessed thyroid hormones and cortisol changes could represent an important tool to evaluate the anabolic and/or catabolic adaptation in response to different functional growth phases in young beef animals. Hence, it is important to continue research investigating the optimal nutritional strategies to improve growing calf performance and health without compromising cost efficiency.

## Figures and Tables

**Table 1:** Composition of concentrate: 0% (control group (CTR)), 10% (low-olive cake group (L-OC)), or 15% (high-olive cake group (H-OC)) olive cake (OC) inclusion.

	CTR	L-OC	H-OC
**Feed composition (% DM)**			
Corn flour	34.00	35.00	35.00
Soybean meal 44%	18.00	15.00	15.00
Corn flakes	13.00	13.00	13.50
Destoned olive cake	-	10.00	15.00
Wheat bran	11.00	4.00	4.00
Barley	10.00	9.00	8.00
Sunflower	7.00	5.00	1.40
Vitamin and mineral mix *	4.00	4.00	3.30
Soybean flakes	2.00	4.00	4.00
Carob	1.00	1.00	0.80
*Saccharomyces cerevisiae*, live yeast	0.40	0.40	0.40
Sodium bicarbonate	0.80	0.80	0.50
Sodium chloride	0.50	0.50	0.50
Sodium propionate	0.30	0.30	0.40
Calcium carbonate	0.30	0.30	0.30
Dicalcium phosphate	0.20	0.20	0.20
**Nutrient composition**			
Dry matter	89.20	88.50	89.00
Crude protein (% DM)	18.50	18.20	18.30
Crude fat (% DM)	5.00	5.40	6.10
Ash (% DM)	5.00	5.10	4.90
Acid detergent fibre (% DM)	8.50	10.60	11.50
Neutral detergent fibre (% DM)	44.30	46.70	45.30
Starch (% DM)	44.00	43.90	43.40
Net energy (UFV/kg of DM) **	1.09	1.08	1.08

* Vitamin E (1500 UI/head/d), selenium (0.30 ppm/head/d), zinc (1000 ppm/head/d). ** The UFV/kg of dry matter intake of concentrate is the unit of energy density according to the INRA feeding system and corresponds to the net energy for meat production (in kcal/kg)/1760.

**Table 2:** Means ± standard deviation for the different groups and model R^2^ and *p*-values for all the included factors (in bold when statistically significant). TSH: thyroid-stimulating hormone, T_3_: total triiodothyronine, fT_3_: free triiodothyronine, T_4_: total thyroxine, fT_4_: free thyroxine.

Sex	Heifers						Bulls												
Time of Sampling	Day 56			Day 147			Day 56			Day 147			*p*-Values						
Diet	CTR	L-OC	H-OC	CTR	L-OC	H-OC	CTR	L-OC	H-OC	CTR	L-OC	H-OC	Diet	Time	Sex	Diet × Time	Diet × Sex	*T*_0_	R^2^
Cortisol (mg/dL)	2.87 ± 0.81	2.97 ± 1.65	3.77 ± 1.13	5.69 ± 2.59	7.23 ± 1.57	5.48 ± 1.36	2.75 ± 1.99	2.37 ± 1.44	2.64 ± 1.26	2.44 ± 1.25	1.87 ± 1.02	2.78 ± 1.05	0.6279	**0.0002**	**0.0003**	0.5246	0.1232	0.1565	0.41
TSH (ng/mL)	0.16 ± 0.06	0.16 ± 0.04	0.18 ± 0.05	0.21 ± 0.13	0.14 ± 0.09	0.20 ± 0.09	0.19 ± 0.07	0.21 ± 0.13	0.21 ± 0.12	0.19 ± 0.07	0.16 ± 0.10	0.16 ± 0.11	0.6015	0.6814	0.6786	0.4188	0.7977	0.2033	0.06
T_3_ (ng/dL)	131.88 ± 15.92	100.35 ± 24.29	111.78 ± 19.03	83.56 ± 20.56	119.01 ± 21.33	91.68 ± 25.15	86.83 ± 10.76	98.85 ± 10.65	93.50 ± 20.78	138.63 ± 11.26	139.38 ± 23.65	120.13 ± 14.49	0.4028	**0.0257**	0.1223	**0.0470**	0.6459	0.0735	0.18
fT_3_ (pg/mL)	3.38 ± 0.36	2.88 ± 0.35	2.96 ± 0.44	2.26 ± 0.47	3.32 ± 0.91	1.93 ± 0.66	2.81 ± 0.37	3.05 ± 0.41	2.64 ± 0.60	3.03 ± 0.75	2.87 ± 0.77	2.82 ± 0.55	0.4720	0.0618	0.4464	0.1304	0.3643	0.3684	0.18
T_4_ (mg/dL)	8.48 ± 1.64	7.27 ± 1.57	8.70 ± 1.82	8.28 ± 1.87	8.61 ± 1.98	9.07 ± 2.46	6.70 ± 0.68	7.82 ± 1.74	7.02 ± 1.70	8.85 ± 1.43	9.17 ± 1.91	7.21 ± 0.70	0.9077	**0.0133**	0.1387	0.4397	0.0566	**0.0406**	0.21
fT_4_ (ng/dL)	2.63 ± 1.01	2.43 ± 0.47	2.46 ± 0.41	2.15 ± 0.52	3.41 ± 0.87	2.59 ± 1.38	1.74 ± 0.45	2.14 ± 0.41	2.03 ± 0.50	2.75 ± 0.44	2.85 ± 0.98	3.05 ± 0.43	0.6350	**0.0001**	0.4876	0.2340	0.1416	**<0.0001**	0.38

**Table 3:** Least-squares means and standard errors for circulating concentrations of cortisol, thyroid-stimulating hormone (TSH), total and free triiodothyronines (T_3_, fT_3_), and thyroxines (T_4_, fT_4_).

	Day 0	Day 56	Day 147
Cortisol (mg/dL)	2.84 ± 0.13	2.90 ± 0.25	4.25 ± 0.25
TSH (ng/mL)	0.14 ± 0.01	0.18 ± 0.05	0.29 ± 0.05
T_3_ (ng/dL)	117.04 ± 2.99	103.86 ± 3.59	115.4 ± 3.59
fT_3_ (pg/mL)	3.68 ± 0.08	2.95 ± 0.09	2.71 ± 0.09
T_4_ (mg/dL)	5.98 ± 0.19	7.67 ± 0.24	8.53 ± 0.24
fT_4_ (ng/dL)	2.04 ± 0.06	2.24 ± 0.1	2.8 ± 0.1

## Data Availability

The raw datasets generated and/or analysed during the current study are available from the corresponding author on reasonable request.

## Supplementary Materials

The following supporting information can be downloaded at: https://www.mdpi.com/article/10.3390/ani13132120/s1, Table S1: Least-squares means ± standard error and *p*-values obtained from the model for the different dietary treatments. TSH: thyroid-stimulating hormone, T_3_: total triiodothyronine, fT_3_: free triiodothyronine, T_4_: total thyroxine, fT_4_: free thyroxine.
