# Supplementary material for: Effects of Dietary Enrichment with Olive Cake on the Thyroid and Adrenocortical Responses in Growing Beef Calves
Source: Animals (Basel). 2023 Jun 26;13(13):2120. doi: 10.3390/ani13132120 (PMC10339893; doi:10.3390/ani13132120)
Supplement: Supplementary file 1 [file animals-13-02120-s001.zip › animals-2449822-supplementary.pdf]

Table S1. Least square mean ± standard error and p-values obtained from the model for the different dietary treatments. TSH: thyroid-stimulating hormone, T_3: total triiodothyronine, fT_3: free triiodothyronine, T_4: total thyroxine, fT_4: free thyroxine.

	**CTRL**	**L-OC**	**H-OC**	**p-value**
**Cortisol (mg/dl)**	2.82 ± 0.43	2.65 ± 0.43	3.22 ± 0.43	0.6279
**TSH (ng/ml)**	0.21 ± 0.1	0.2 ± 0.1	0.14 ± 0.1	0.8496
**T_3 (ng/dl)**	110.12 ± 6.24	98.13 ± 6.27	103.34 ± 6.23	0.3519
**fT_3 (pg/ml)**	3.09 ± 0.16	2.95 ± 0.16	2.81 ± 0.16	0.3392
**T_4 (mg/dl)**	7.52 ± 0.42	7.69 ± 0.42	7.78 ± 0.42	0.9268
**fT_4 (ng/dl)**	2.17 ± 0.17	2.37 ± 0.17	2.18 ± 0.17	0.5846
